# A versatile computerized polarograph, polarographic sample changer and data-acquisition system: applications in electrode mechanism studies

**DOI:** 10.1155/S146392468700004X

**Published:** 1987

**Authors:** H. H. J. L. Ploegmakers, M. J. M. Mertens, R. J. Driebergen, W. J. van Oort, A. Dirksen, W. van der Horst, A. C. C. van Til, R. van Trigt

**Affiliations:** Department of Analytical Pharmacy Subfaculty of Pharmacy State University of Utrecht Catharijnesingel 60 Utrecht 3511 GH The Netherlands


Journal of Automatic Chemistry/*Journal of Clinical Laboratory Automation*, Vol. 9, No. 1 (January–March 1987), pp. 15–24

### A versatile computerized polarograph, polarographic sample changer and data-acquisition system: applications in electrode mechanism studies

**H. H. J. L. Ploegmakers, M. J. M. Mertens, R. J. Driebergen, W. J. van Oort, A. Dirksen*, W. van der Horst*, A. C. C. van Til* and R. van Trigt***

*Department of Analytical Pharmacy, Subfaculty of Pharmacy, State University of Utrecht, Catharijnesingel 60, 3511 GH Utrecht, The Netherlands*

### Introduction

The mechanism of action of various anticancer drugs may be explained via preceding biochemical redox activation. It has been suggested that mitomycins and anthracyclines, two well-known classes of quinoid cytostatistics, have to be reduced to unfold their cytotoxic properties, whereas the parent compounds, in quinoid forms, are not active, Moore [1].

Consequently, there may be a relationship between the electrochemial properties of these compounds and their biochemical activation process. In order to study this possible relation and the influence of substitution, a wide variation of analogues and derivatives should be synthesized and analysed electrochemically. To examine the electrochemistry of a compound and to elucidate its reduction mechanism, two features of organic polarography have been proved to be very useful: half-wave potential-pH and limiting current-pH relationships, Zuman [2].

The mathematics of these relationships are relatively well developed, Bond [3]; several electrochemical parameters can be deduced from these relationships. Investigations of the electrochemical behaviour of a particular compound over a wide pH range is the essential part of the procedure. A large series of analysis has to be carried out comprising d.c. polarographic recordings of sample and blank solution over a potential range of −1500 mV at a scan rate of −2 mV/s at intervals of 0·3 pH-unit or less, and 10 min purging with nitrogen of each solution. Each analysis requires about 35 min measuring time. The registration of a complete pH-profile varying from for example pH = 2 to pH = 13 with intervals of 0·3 pH-units, including samples of equal pH but different buffer composition, requires about 40 samples, consuming approximately 24 h of analysis. The study on quinones, mentioned before, comprises 60–70 analogues and derivatives. As such a procedure is rather time-consuming, it was decided to develop a system in which the large series of time-consuming measurements can be performed fully unattendedly.

* Engineering Division.

In other disciplines of analysis several systems have been described, in electrochemical analysis only a few attempts have been made (Cooley [4]). Some examples of sample changers are described by Hiebert *et al.* [5] and Hodgson and Brown [6], which sample changer is combined with an alpha-particle detector. Pinglot and Pourchet [7] measure the beta-activity of series of polar ice cap samples, Jost *et al.* [8], Beneson *et al.* [9] and Marrum and Schober [10] describe the use of sample changers equipped with gamma radiation detectors, Potts and Hussey [11] apply automated neutron activation analysis, Holland and Medrud [12] X-ray diffraction and Riedel [13] measures the half-lifetime of several radionuclides. Bakker *et al.* [14] describe an automatic sample changer for infrared spectrometry, programmable for repetitive scanning over a selected wavelength range.

Some of the authors apply a (micro)computer to control the system and perform data-processing [8,10 and 12].

In clinical analysis several large systems, including sample changers, are available. These sample-changers are expensive commercially available systems, designed for processing ready-for-use samples, whereas the application (X-ray-, gamma-ray- and UV detection) does not require extensive software; and the PAR 374 Polarographic Analyzer proved not sufficiently reliable for routine analysis.

In this paper, the development of a low-cost fully computerized polarographic sample changer and data-acquisition system will be described.

Originally, the system was intended to be used as automatic measuring and storage system of polarographic recordings at several pH-values. During a polarographic analysis most of the time is consumed by deaeration of the solution by nitrogen gas; therefore automation including a microcomputer offers the possibility to perform data-handling, for example half-wave potential and limiting current calculation during purging of the solution next to be analysed.

The system has many applications – for multi-sample analysis in equilibrium, stability and environmental studies for instance.

As an example, the analysis and the data-processing of the polarographic analysis of a simple compound, 2-amino-1,4-naphthoquinone, will be demonstrated, showing unsampled d.c. and sampled d.c. polarograms, blank subtraction, first derivative, half-wave potential calculation, the half-wave potential-pH plot, the slope of the E1/2-pH plot and the limiting current-pH plot. As an example of a more complex compound, the half-wave potential-pH plot and the limiting current-pH plot of 2,5-bis(1-aziridinyl)-1,4-benzoquinone will be determined and presented.

### Experimental

The heart of the sample changer, data acquisition and handling system (figure 1) is an Apple IIe computer, equipped with 64 kbytes of RAM. To this system the following peripherals have been added: two 125 kbytes Apple 5·25 in floppy disk drives, an Apple monitor, an Itoh 8510 parallel printer, a Kronemuis two-channel 12-bits D/A converter, an unipolar home-made four-channel, a 12-bits A/D converter (van den Belt [15]), a California Computer Systems model 7440A programmable timer module, a Kronemuis APL 13 graphic printer interface with screen dump facilities, a California Computer Systems 7720 peripheral interface adaptor (PIA) module, an IBS AP-30 64-channel I/O device (quad 6522 versatile interface adaptor [VIA]), a Metrohm 655 Dosimat microprocessor-controlled piston buret equipped with an anti-diffusion tip, a Radiometer PHM 64 research pH-meter equipped with a Ingold LOT 401 glass/reference electrode, Salm & Kipp Thermomix 1420 thermostat, a Metrohm 641 VA-Detector potentiostat, a Houston Instrument Microscribe 4500 digital controlled analogue strip-chart recorder and a Hewlett-Packard 7470 A plotter.

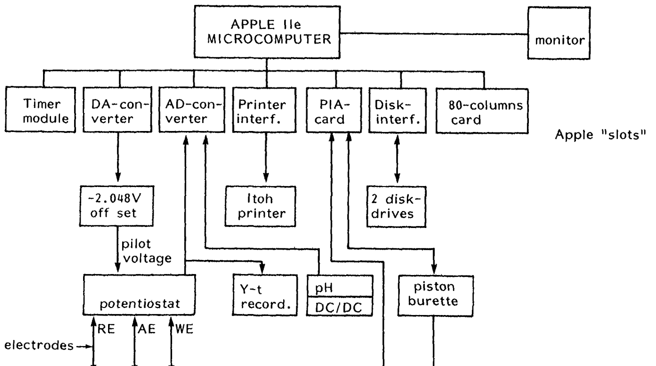

*Figure 1. Schematic diagram of the computerized polarographic sample changer and data acquisition system.*

A home-made computer-controlled sample changer (figure 2) completes the system.

### *Description of the sample changer*

The sample changer (see figure 2) consists of a mechanical and an electronic part, controlled by dedicated software.

*The mechanical part:* the sample positioner consists of a platter (diameter 29 cm), containing 40 holes; in each hole a 22 ml test-tube, each filled with a different buffer solution. The platter is rotated by an alternating current motor, M, equipped with a decelerator. On the axis of the motor light-interrupting wings have been attached: the position of the platter is controlled by opto-couplers.

If a tube is in the right position, a stainless-steel needle is lowered pneumatically into the solution; the buffer solution is then transferred to the polarographic cell by an LKB peristaltic pump 1 (see figure 2).

The Dosimat piston burette is used for dosage of the sample solution. Originally, the burette was activated by the microcomputer by means of a PIA. After introducing the computer-controlled recorder into the system, requiring more logic lines, the PIA-module was replaced by a quad VIA module.

The peristaltic pump 2 is used to rinse the cell.

A transistor-transistor-logic (TTL)-adapted Metrohm E 354 Polarographic Stand is used as a mercury drop dislodge mechanism, controlled by a logic output of the Apple game connector.

The content of the covered Perspex polarographic cell is about 10 ml. A water jacket allows application of a thermostat (20 °C). A software-controllable valve in the bottom enables removal of its content.

The electrodes and the nitrogen inlets and outlets are placed in the cover of the cell. The nitrogen flow through and over the solution is directed by two valves, also regulated by the VIA. The reference electrode is a home-made Ag/AgCl (3 M KCl) electrode with a Vycor glass tip; the auxiliary electrode consists of a platinum wire.

*The electronic part:* the electronic part consists of a 6809 microprocessor, a homemade monitor containing EPROM, internal electronics and I/O-interfacing.

The monitor program controls the position of the electric motor, the optocouplers and the feedback to the motor, the nitrogen valve for the needle positioning and the I/O-interfacing with the host computer. After receiving a logical zero from the host, the needle is elevated, the platter is rotated one position and the needle is lowered; next a 'ready' pulse is sent back to the host. In this way it is impossible to damage the platter or needle by giving wrong control commands.

The four-phase stepper motors of the LKB peristaltic pumps are controlled by a host-computer generated square wave signal (frequency limit 150 Hz, duty cycle 50%).

The polarographic cell is equipped with a combi-electrode, to monitor the pH of the solution. The output of the pH meter is connected to an input of the A/D-converter.

### *Software*

The software exists of eight Applesoft Basic programs and three subroutines written in 6502 assembly language

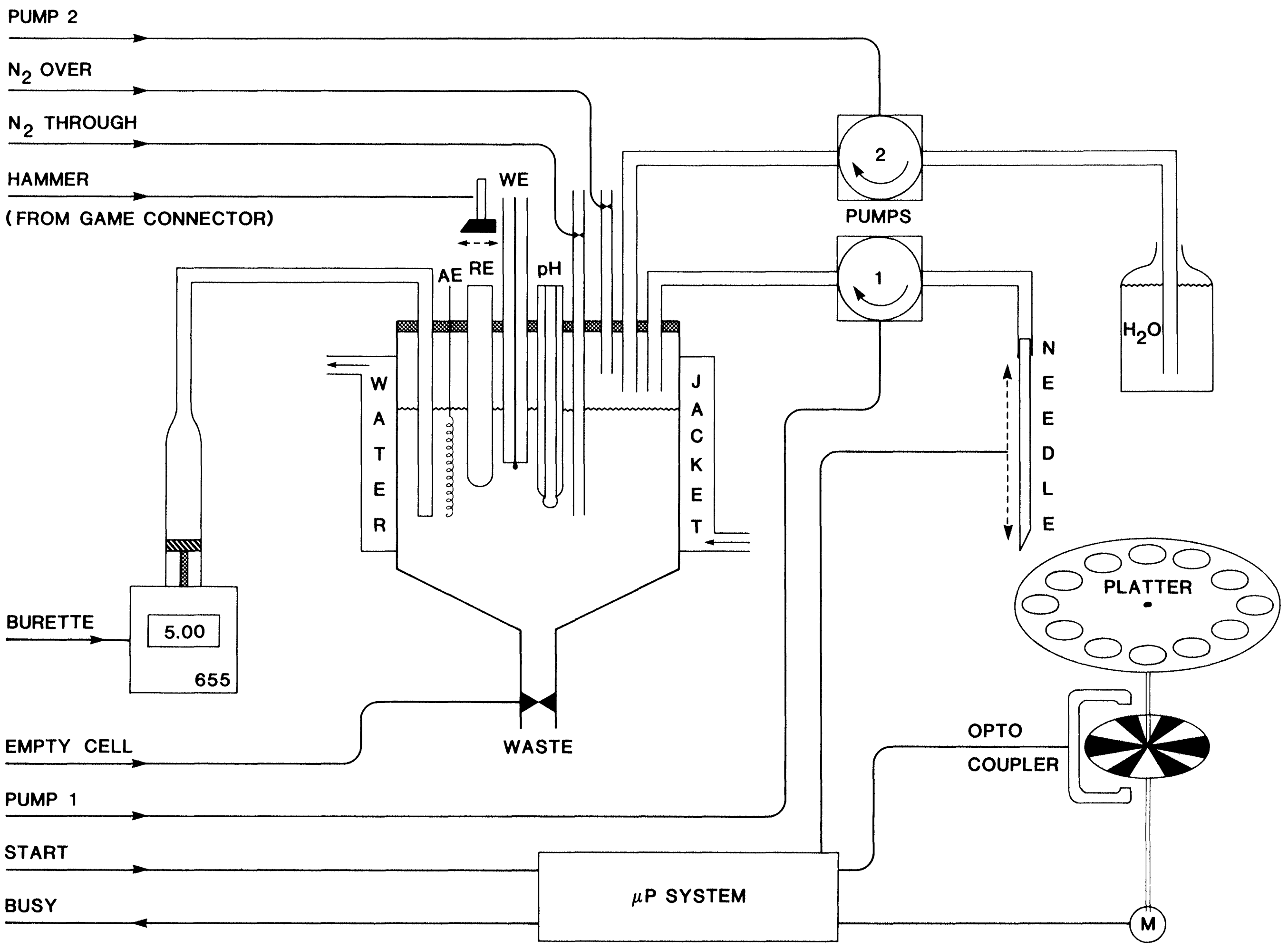

*Figure 2. Schematic diagram of the sample changer.*

(table 1). A listing of the programs can be obtained from the authors.

*Recording of the polarographic curve:* the flow-chart (figure 3) indicates that the applied electrochemical method is not the classical form of d.c.-polarography but a method which may be described as sampled staircase d.c.-polarography.

Electrochemical parameters like initial voltage, scan range and scan rate, droptime and buffer- and sample volume are user-defined. Changing these parameters allows the operator to perform d.c. polarography, as well as cyclic voltammetry. The latter method proved to be useful for stability of compounds by monitoring automatically the decrement of the parent peak (or the increment of new peaks) of a compound as a function of time at different pH-values.

If desired, a paper-copy of blank and sample d.c.-polarograms can be obtained using the digitally controlled analogue Y-t recorder, connected directly to the potentiostat output. The paper-copy, recorded in this way, shows the original, non-sampled d.c. polarograms (figure 4).

Measuring dilute samples and using analogue/digital converters induce noise in the measured currents, and this cannot be neglected. Because the direction of the noise vector changes continuously, this noise can be eliminated to a large extent by averaging a series of measurements. If, at 2 s after drop dislodging, the capacity current reaches an almost constant and large value, 16 current measurements are performed, as fast as possible after each other; the values are averaged. Because the actual current measurements should not influence the drop-timing, the program is written in assembly language. In this way, the 16 measurements can be performed and averaged within half a millisecond, which is negligible compared with the drop-time.

After recording the polarograms of the blank and the sample, both polarograms are stored on disk.

*Automatic adaptation of the starting potential:* in acid and neutral solutions, mercury is oxidized at potentials more positive than +300 mV versus the Ag/AgCl reference electrode. In more alkaline solutions and/or solutions containing, for example halides, oxidation proceeds at less positive potentials (Meites [16]), disturbing the polarograms and consequently hampering the calcula-

*Table 1. Program function.*

| Language | Name | Function |
|---|---|---|
| Applesoft Basic | LOADER | to move basic program space to memory location $4000 |
| Applesoft Basic | SET UP | to load machine language routines; to load parameters; deaeration of the first buffer solution; to call MEASPROG |
| Applesoft Basic | MEASPROG | to call MCODE; to measure pH of the buffer; to record blank polarogram; to mix buffer with sample; to call MCODE; to record a polarogram of the sample solution; to store both polarograms on disk; to start deaeration of the next buffer; to call CALCPROG |
| Applesoft Basic | CALCPROG | to call FILTCODE to filter both polarograms; blank subtraction; to calculate halfwavepotential and limiting current of every wave; return to MEASPROG |
| Applesoft Basic | TABLEMAKER | to table the calculated E1/2 values and plot these vs. pH |
| 6502 Assembly | MCODE | to record a polarogram using data averaging |
| 6502 Assembly | FILTCODE | parabolic filter |
| 6502 Assembly | PUMPCODE | peristaltic pump control |
| Applesoft Basic | E/pH-PLOT | to plot E1/2 vs. pH |
| Applesoft Basic | i/pH-PLOT | to plot ilim vs. pH |
| Applesoft Basic | SLOPE-PLOT | to calculate and to plot the slope of the E1/2-pH relationship |

tions. In more alkaline solutions, it is necessary to decrease the initial voltages. In addition, the system was particularly for experiments with quinoid anti-cancer drugs. Many of these compounds show chemical degradation in alkaline solutions (Stevens [17] and Taylor [18]). Because of the relatively slow scan rate, substantial amounts of unstable substances may already be present in the alkaline solution before reaching the half-wave potential. By decreasing the difference between initial voltage and half-wave potential, these undesirable chemical degradations are limited.

To solve both problems: at pH-values lower than an user-defined pH, the polarograms are recorded from the same starting potential; at higher pH-values, a second initial voltage is chosen automatically.

*Half-wave potential calculation:* to determine the number of waves in a polarogram and their half-wave potentials, the 'pseudo-first-derivative' of the blank corrected polarogram is determined and the number of peaks is calculated. However, the derivative curve shows a highly noisy character, so curve filtering is necessary. Because of the favourable properties of a parabolic filter (Ploegmakers [19]), this subroutine is implemented in the software. The version described is written in Applesoft Basic, resulting in a relativily long execution time. To perform all calculations during the period of purging with nitrogen of the next blank solution, an 11-point parabolic filter program, written in machine language (FILTCODE) is inserted, achieving a time gain of about 80%. The effect of FILTCODE is showed in figures 5(*b*)–(*d*). Testing the program on the same curve as described earlier (Ploegmakers [19]) it is shown that it has the same favourable properties as the Basic program but that the curve is barely affected.

The construction and calculation of the half-wave potential (figure 6) has been described earlier (Ploegmakers [19] and Meites [20]).

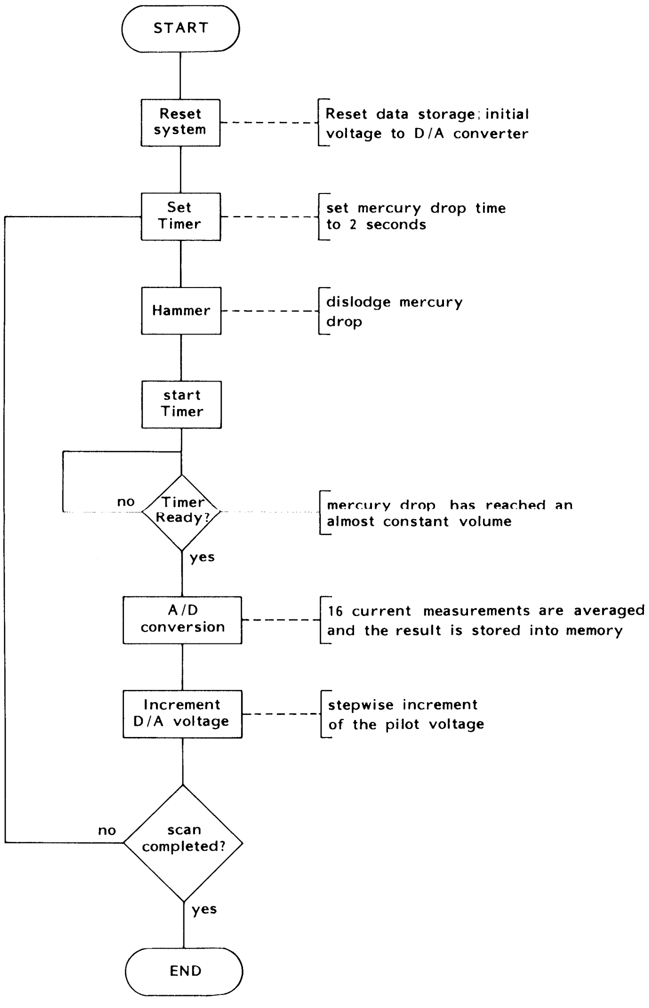

*Figure 3. Flow-chart of the data acquisition.*

*Thallium correction:* during long series of experiments the potential of the reference electrode may change, for example due to diffusion processes: a correction has to be made for this error.

Because thallium ions are reduced in a well-defined reduction wave with a reproducible half-wave potential of −455 mV, independent of pH, its half-wave potential is used to check and correct the system for deviations of the reference electrode potential. If this thallium correction is urged, the determination of a series of samples starts with the calculation of the half-wave potential of thallium sulphate at pH 7·0. The result is compared with the true value (−455 mV) and the deviation is used to correct all half-wave potentials of the samples automatically.

*Logarithmic analysis:* the equation

$$E = E_{1/2} + RT/\text{alpha}.nF.\ \ln((i_d - i)/i) \qquad (1)$$

derived by Heyrovsky and Ilkovic [21] gives the correlation for each point of the polarogram between potential, half-wave potential, current, limiting current and number of electrons transferred, of a reversible (alpha = 1) reductive electrochemical reaction.

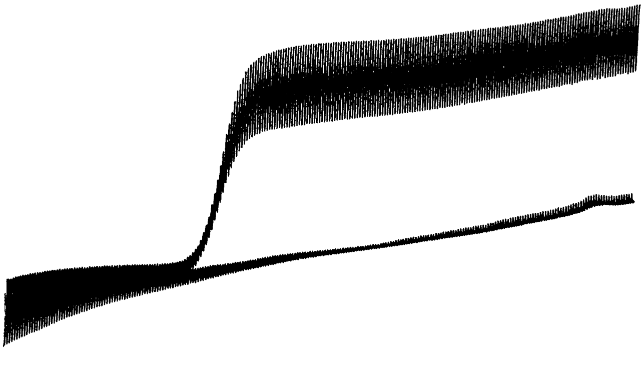

*Figure 4. D.c. polarogram of 2-amino-1,4-naphthoquinone, recorded with the computerized sample changer and data acquisition system. Conditions: concentration: 5·5 . 10–5 M in 0·1 M phosphate buffer pH = 7; initial voltage: 0 V; scan range: −1 V; scan rate: 2 mV/s; current range: 1 μA F.S.; temperature: 25 °C.*

So, by plotting $E$ vs. $\log((i_d - i)/i)$ (2)

a linear relation will be obtained indicating the half-wave potential with the log term is zero and with a slope of 2·303. RT/alpha.*n*F, where *n* is the number of transferred electrons and alpha the transfer coefficient (see figure 7).

For example in the case of the reduction of thallous (III), lead (II) and indic (I)ions the slopes of the log plots are 0·059, 0·033 and 0·023 V, which are in close agreement with the theoretical values which are 0·059, 0·030 and 0·020 V, respectively (Kolthoff [22]). Before the introduction of the microcomputer, these calculations were very time-consuming and tedious.

In the system presented here, all the needed calculation facilities are present and all potentials and the corresponding current values, are stored in an array, so the number *n* or the product *n*.alpha may be determined easily.

*The plot-programs:* the application, mentioned in this paper, requires a lot of polarographic analysis of the same compound at several pH-values. The results at one pH-value, obtained from the data-processing and calculation programs, do not give mechanistic information, rather than the pH-dependencies of E1/2 and limiting current values. As it is obvious to extend the system with options, processing and presenting results, to show these relationships.

The function of the E/pH-PLOT program (figure 8) is to show the relationship between the calculated half-wave potentials of each wave and the corresponding pH-values. For a reversible system, the slope of the E1/2-pH plot at 25°C equals 0·0591.m/n, where m is the number of hydrogen ions participating in the electrode-reaction and *n* the number of electrons transferred in the reaction (Meites [23]).

Because the slope of such a plot may vary within the normal pH-range, due to changing protonation and/or electron uptake, the SLOPE-PLOT program has been written (figure 9). This program calculates the slopes of

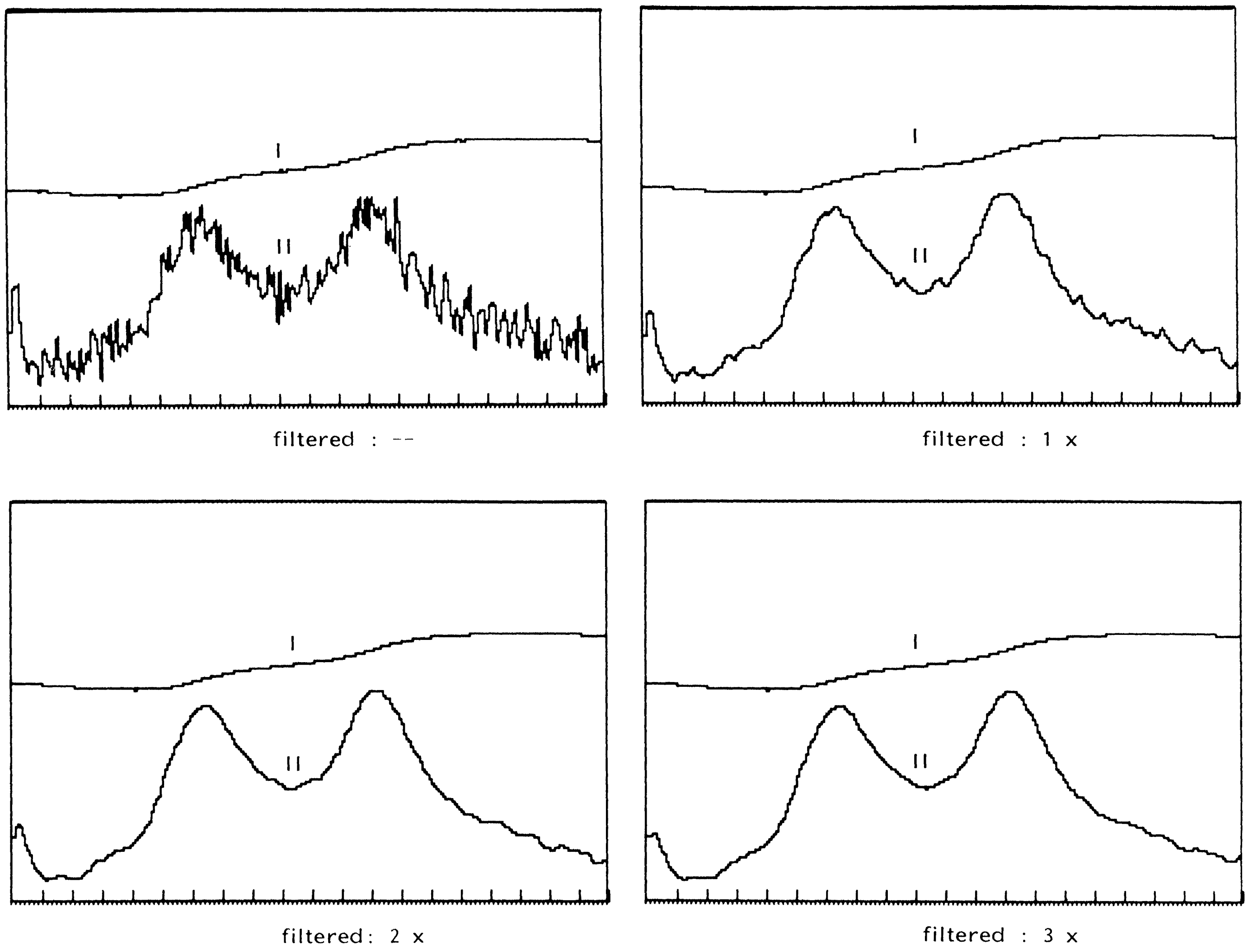

*Figure 5 (a)–(d). The influence of repetitive application of the FILTCODE filter program on d.c. curves (I) and first derivative curves (II) of an oxidative on-line linear sweep voltammogram after HPLC of 13 μg Etoposide (VP 16–213): (a) not filtered; (b) filtered 1x; (c) filtered 2x; (d) filtered 3x.*

the E/pH-plot, using the least squares method. It plots the calculated slopes as a function of pH.

The function of the i/pH-PLOT program (figure 10) is to present the relation between the limiting current of each wave and the pH.

### Results

During testing of the system, interference between the polarographic and the pH-circuit was observed, probably due to interference caused by the potential of the reference electrode of the combi-electrode.

The problem could be solved by inserting an isolating DC/DC transformer (Knick 15A) between the two circuits.

### *Reproducibility*

To investigate the reproducibility of the polarograph and data acquisition system, 20 tubes filled with 0·1 M phosphate buffer pH = 7 and a 0·005 M solution of naphthoquinone are analysed. The results have been mentioned in table 2. It proves that the measurements of pH, limiting current and half-wave potential are sufficient reproducible for our purposes (pH = 6·96; SD = 0·01) (current = 140 nA; SD = 7 nA) (half-wave potential = −141 mV; SD = −2 mV).

### *Half-wave potential versus pH relationship*

In electrode mechanism studies of organic compounds, it is useful to plot the half-wave potential(s) and limiting current(s) as a function of pH.

During electrochemical reduction of many organic substances, hydrogen ions are involved. If the compound is protonated before or after the electrons have been taken up, the process and consequently the half-wave potential will be dependent on the hydrogen concentration. By recording polarograms at several pH values and with small intervals and plotting the half-wave potentials vs. pH, the relationship between E1/2 and pH can be established. Applying the described polarograph, it can be performed automatically.

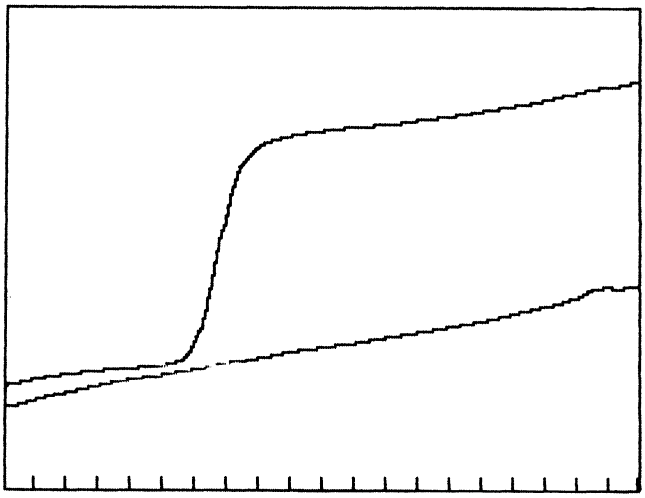

(*a*)

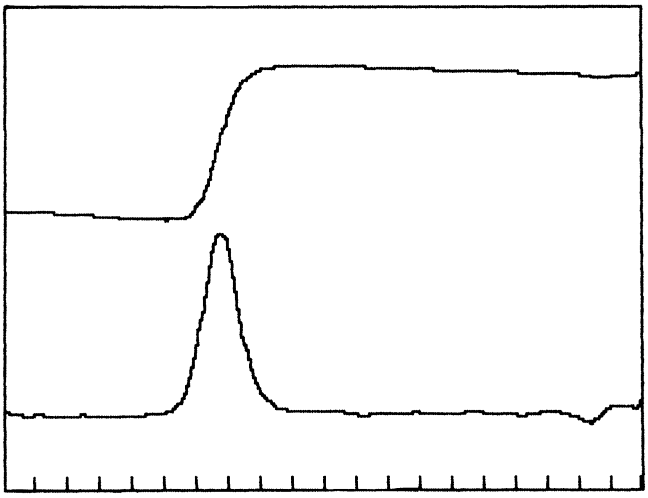

(*b*)

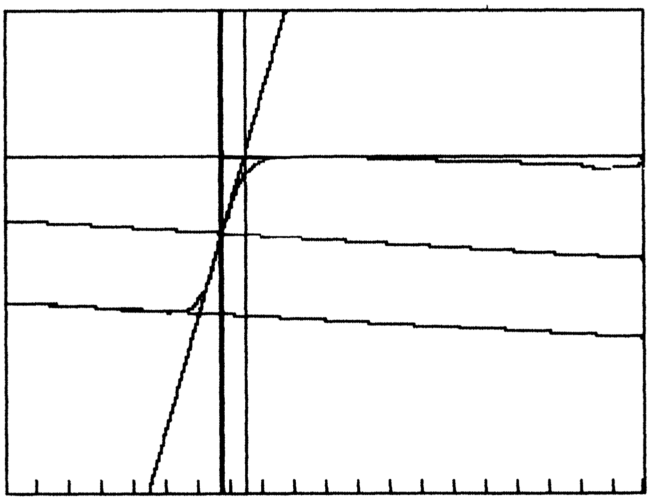

(*c*)

*Figure 6 (a) Polarogram of the blank solution and a solution of 2-amino-1,4-naphthoquinone. (b) Blank-corrected polarogram of 2-amino-1,4-naphthoquinone and its first derivative. (c) Construction of the half-wave potential of blank-corrected polarogram of 2-amino-1,4-naphthoquinone. Conditions: concentration: 5·5 . 10–5 M in 0·1 M phosphate buffer pH = 7; initial voltage: 0 V; scan range: −1 V; scan rate: 2 mV/s; current range: 1 μA F.S.; temperature: 25 °C.*

*Table 2. Testing the reproducibility of the computerized polarographic sample changer and data acquisition system by measuring of the pH, limiting current and half-wave potential. Conditions: 5 ml 0·1 M phosphate buffer pH = 7·0; 50 μl 0·005 M naphthquinone added; Sensitivity: 1 μA F.S.; Scan rate: 2 mV/s; Drop time: 2 s; Initial voltage: +200 mV; Scan range: −1 V.*

| Nr. | pH | i (nA) 1 | E1/2 1 | (mV) (not corrected) |
|---|---|---|---|---|
| 1 | 6·97 | 142 | −145 | |
| 2 | 6·97 | 132 | −145 | |
| 3 | 6·99 | 137 | −145 | |
| 4 | 6·99 | 134 | −141 | |
| 5 | 6·98 | 132 | −141 | |
| 6 | 6·98 | 132 | −141 | |
| 7 | 6·97 | 135 | −141 | |
| 8 | 6·97 | 140 | −141 | |
| 9 | 6·97 | 157 | −141 | |
| 10 | 6·96 | 137 | −141 | |
| 11 | 6·96 | 147 | −141 | |
| 12 | 6·96 | 142 | −141 | |
| 13 | 6·96 | 145 | −141 | |
| 14 | 6·96 | 145 | −141 | |
| 15 | 6·96 | 143 | −141 | |
| 16 | 6·95 | 132 | −141 | |
| 17 | 6·96 | 154 | −145 | |
| 18 | 6·95 | 144 | −141 | |
| 19 | 6·95 | 134 | −141 | |
| 20 | 6·96 | 144 | −141 | |

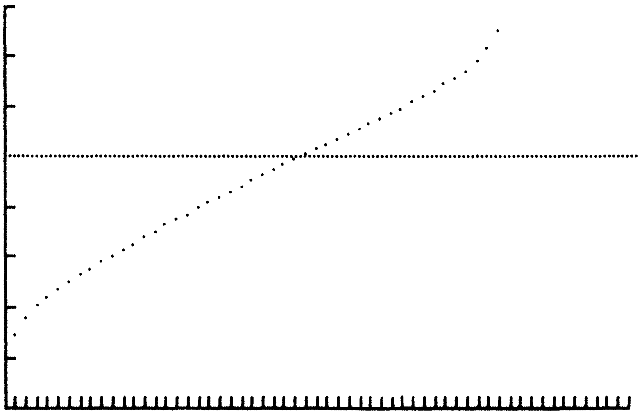

*Figure 7. The logarithmic analysis of the polarographic wave of 2-amino-1,4-naphthoquinone and the determination of the half-wave potential. Conditions: concentration: 5·5 . 10–5 M in 0·1 M phosphate buffer pH = 7; initial voltage: 0 V; scan range: −1 V; scan rate: 2 mV/s; current range: 1 μA F.S.; temperature: 25 °C.*

The E/pH-PLOT program enables the graphical reproduction of the relationships.

As an example, the half-wave potential-pH plot of 2-amino-1,4-naphthoquinone is showed (figure 8). There proves to be a linear correlation between half-wave potential and pH from pH = 4 up to pH = 10; at higher pH values the reaction becomes independent of the pH due to decreased protonation of the formed dianion, whereas the inflexion-point in the E1/2-pH-plot observed

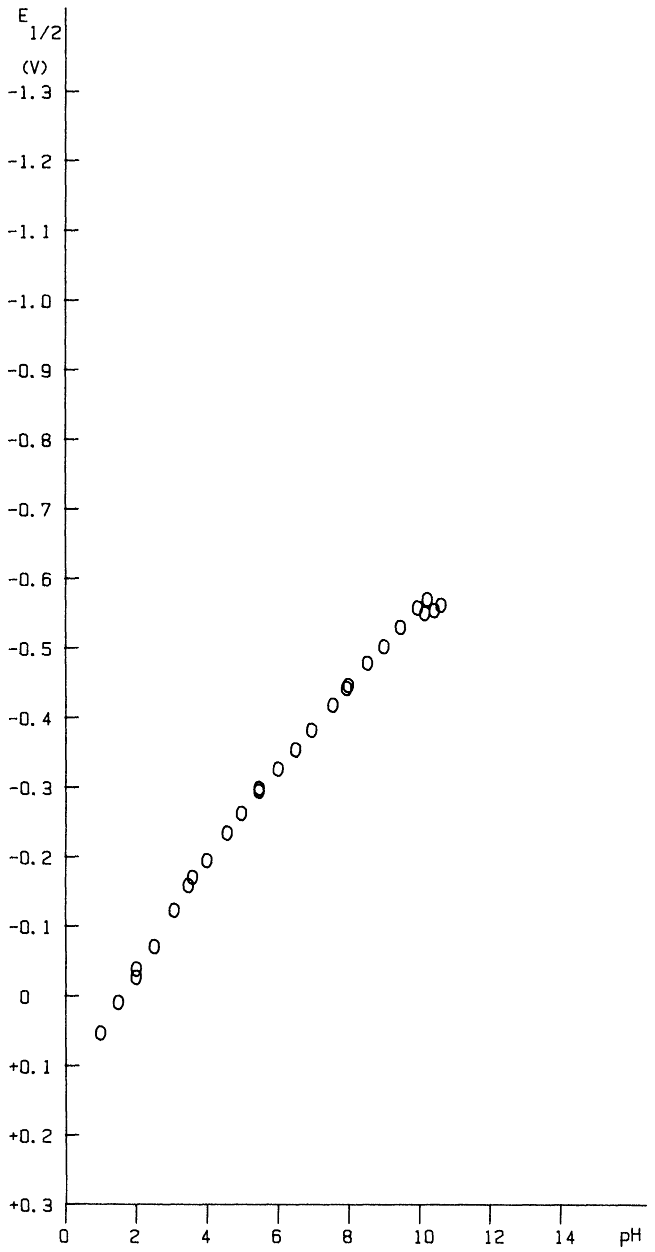

*Figure 8. The relation between half-wave potential of 2-amino-1,4-naphthoquinone and pH. Conditions: concentration: 5·5 . 10–5 M in 0·1 M buffer; scan range: −1 V; scan rate: 2 mV/s; current range: 1 μA F.S.; temperature: 25 °C.*

at pH = 4 corresponds with the pKa of the amino function of the formed hydroquinone. Due to the protonation of this function at pH < 4, the ratio of m/n becomes 3/2 and a decreased slope of −60 to −90 mV/pH-unit is observed (den Hartigh [24]). However, to determine such inflexion points accurately, small pH-intervals are required.

The SLOPE-PLOT program has been designed to determine such inflexion points. In this program, the slope of the linear regression line is calculated from a number of successive half-wave potentials, for example the first up to the fourth, the second up to the fifth and so on. The slope of such a line is plotted as a function of the pH of the last point of the bunch.

Figure 9 is an example of such a plot. It shows that at pH-values < 4, the slope is −90 mV/pH-unit, decreasing to −60 mV/pH-unit at pH = 8 whereas the slope becomes constant at higher pH-value.

*Limiting current versus pH relationship*

Usually the height of the diffusion current is determined by the rate of diffusion of the electroactive species to the surface of the electrode. However, phenomena such as homogeneous and heterogeneous chemical reactions, adsorption and catalytic reactions, affect the height of the limiting current as well. In absence of these (complicating) phenomena, usually a diffusion-controlled current is observed, linearly proportional to the concentration of the electroactive species. From the i–E curves quantitative information on the composition of the solution can be obtained. As an example, the limiting current-pH plot of 2-amino-1,4-naphthoquinone is showed (figure 10).

In organic polarography often a strong dependence of for example the half-wave potential, the wave height and the shape of the wave on pH will be observed. Acid-base

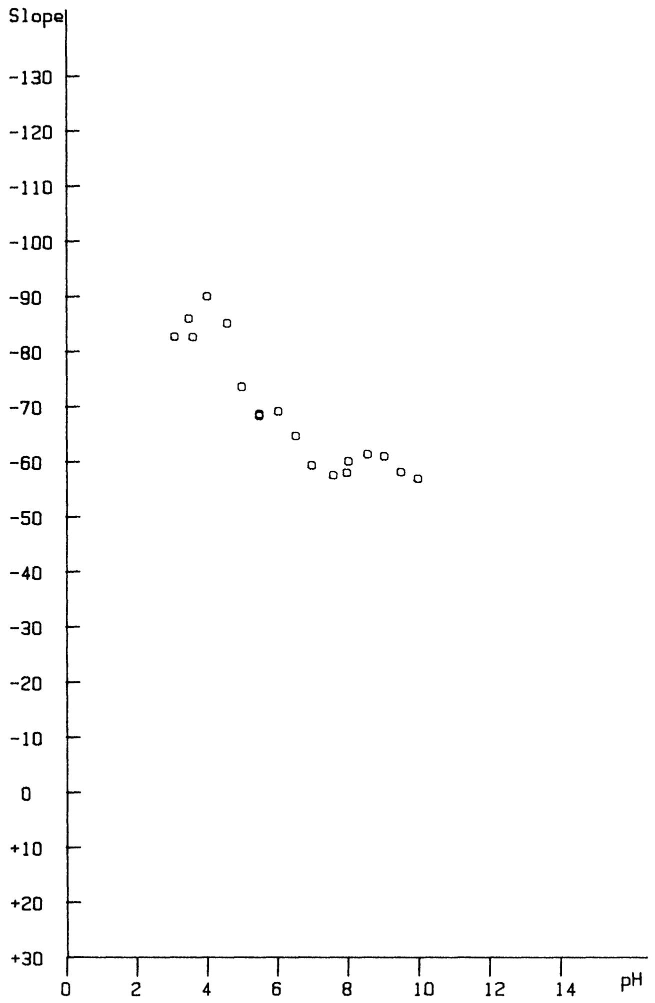

*Figure 9. The relation between slope of the E1/2-pH relationship of 2-amino-1,4-naphthoquinone and pH. Conditions: concentration: 5·5 . 10–5 M in 0·1 M buffer; scan range: −1 V; scan rate: 2 mV/s; current range: 1 μA F.S.; temperature: 25 °C.*

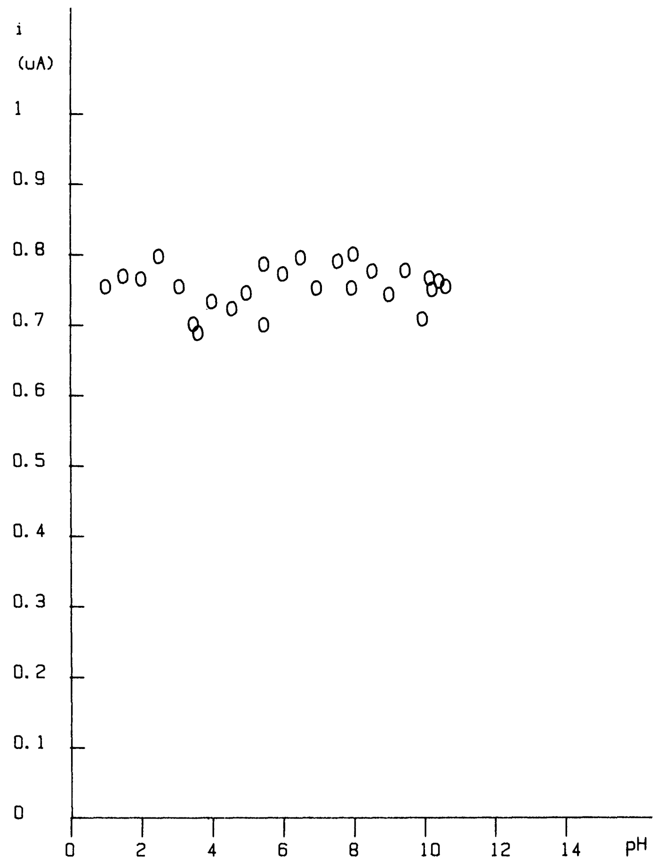

*Figure 10. The relation between limiting current of 2-amino-1,4-naphthoquinone and pH. Conditions: concentration: 5·5 . 10–5 M in 0·1 M buffer; scan range: −1 V; scan rate: 2 mV/s; current range: 1 μA F.S.; temperature: 25 °C.*

equilibria, taking place in the bulk of the solution or in the vicinity of the electrode surface and preceding the electrode process affect the concentration of electro-active species directly. Furthermore, the chemical stability of the parent compound and of the generated product may also depend (strongly) on the pH. Consequently, there will be changes in polarographic curves with pH.

To elucidate the complex reduction mechanism of 2,5-bis(1-aziridinyl)1,4-benzoquinone, detailed E1/2/pH (figure 11) and ilim/pH (figure 12) plots obtained from sampled d.c. polarographic experiments are required and have to be combined with results obtained with other electrochemical techniques like cyclic voltammetry, differential pulse polarography and constant potential electrolysis.

The complete interpretation of the electrode mechanisms, illustrated in figures 11 and 12 will be published elsewhere (Driebergen [25]), as a part of an investigation of a large series of quinoid anti-cancer compounds.

### Discussion

The computerized polarographic sample changer and data acquisition system described is a reliable instrument, capable of automatically performing complete polarographic analysis and decreasing the analysis time considerably.

At the moment of writing, the system has been in operation for one year without any technical failures.

The application of commonly available computer hardware and homemade software, written in Applesoft Basic and 6502 assembly language, allows copying and adapting the system for other users. The flexibility of the user-defined electrochemical parameters like the scan-rate, not only offers the possibility of performing d.c. polarography, but also cyclic voltammetry; the latter enables research on stability of drugs in solutions.

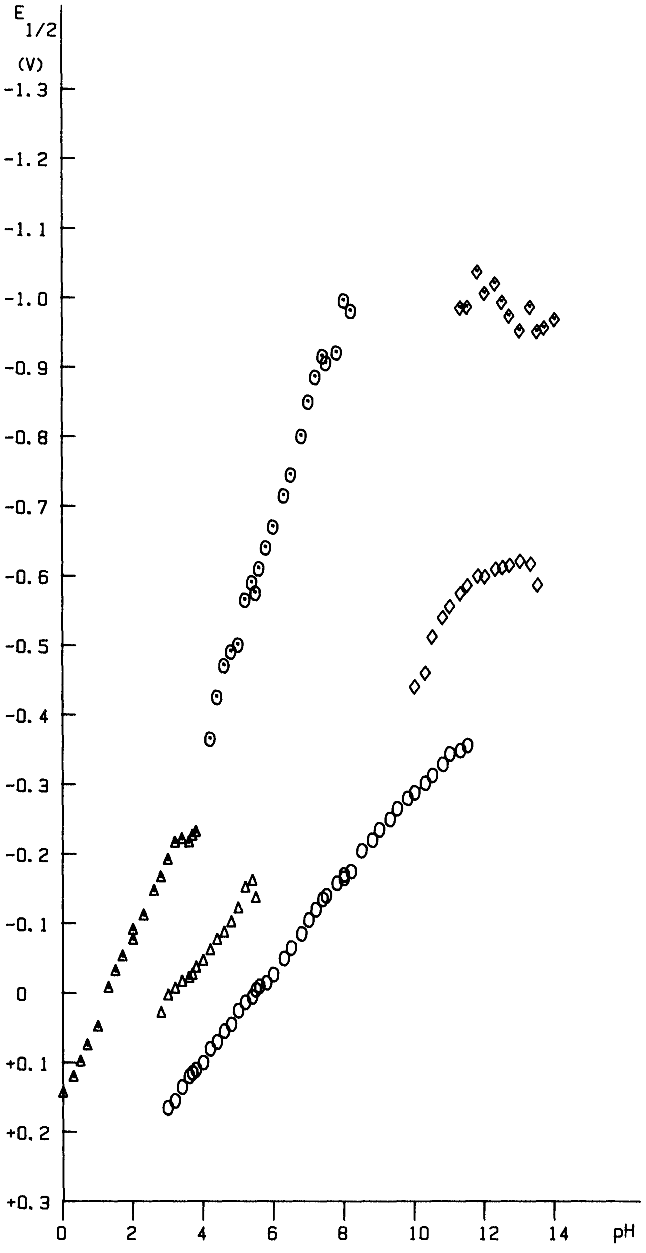

*Figure 11. The relation between half-wave potential of 2,5-bis(1-aziridinyl)-1,4-benzoquinone and pH. Conditions: concentration: 5·0 . 10–5 M in 0·1 M buffer; scan range: −1 V; scan rate: 2 mV/s; current range: 1 μA F.S.; temperature: 25 °C.*

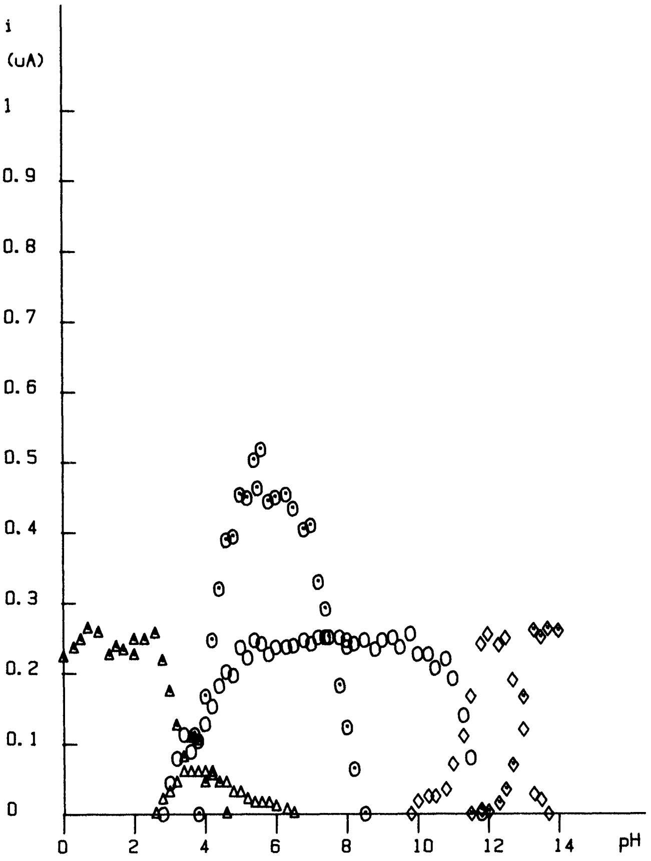

*Figure 12. The relation between limiting current of 2,5-bis(1-aziridinyl)-1,4-benzoquinone and pH. Conditions: concentration: 5·0 . 10–5 M in 0·1 M buffer; scan range: −1 V; scan rate: 2 mV/s; current range: 1 µA F.S.; temperature: 25 °C.*

The instrument has the following features: sample changing, pH measurement and storage, purging with nitrogen, recording and storage of the polarograms of blank and sample solution, recording of unsampled polarograms, correction of spikes caused by prematurely dislodged mercury drops, curves filtering, blank subtraction, logarithmic analysis, thallium correction, half-wave potential and limiting current calculation. When all measurements have been done, the TABLEMAKER program may be used to present the acquired data in a tabular and graphical layout.

Application of automatic calculations on non-ideal curves may lead to faulty or undesirable results. The software has a limited ability to remove faulty results by comparing them with user-supplied limits of the calculated data. However, despite its reliability, each analyst prefers to perform a visual check of the results after each run. Due to the graphical presentation of the data, this can be performed quite easily.

After rejecting possible faulty results, the half-wave potentials and limiting currents are plotted versus pH and the slopes of the E1/2/pH relation are calculated.

### Conclusions

The reliability and reproducibility, presented here, results into automatically obtained plots (figures 11 and 12) which are considerably better interpretable than hand-made plots.

The speed of the system enables implementation of electrode mechanism studies into biochemical and other research.

### References

1. Moore, H. W. and Czerniak, R., *Medicinal Research Reviews*, **1** (1981), 249.
2. Zuman, P. and Perrin, C. L., *Organic Polarography* (Interscience Publishers, New York, 1969).
3. Bond, A. M., *Modern Polarographic Methods in Analytical Chemistry* (Marcel Dekker, New York, 1980), p. 84.
4. Cooley, R. E., Stevenson, C. E. and Rickard, E. C., *Journal of Automatic Chemistry*, **2** (1980), 60.
5. Hiebert, R., Ide, H. and Boyd, H., *Health Physics*, **32** (1976), 311–313.
6. Hodgson, L. M. and Brown, D. A., *Journal of Radioanalytical Chemistry*, **54** (1979), 211.
7. Pinglot, J. F. and Pourchet, M., *Nuclear Instruments and Methods*, **166** (1979), 483.
8. Jost, D. T., Krahenbuhl, U. and von Gunten, H. R., *Nuclear Instruments and Methods*, **197** (1982), 365.
9. Beneson, A., Mersel, M., Pinson, A. and Heller, M., *Analytical Biochemistry*, **96** (1979), 402.
10. Marrum, M. T. and Schober, H. A., *G-I-T Fachzeitschrift fur das Laboratorium*, **21** (1977), 575.
11. Potts, P. J. and Hussey, R., *Journal of Radioanalytical Chemistry*, **78** (1983), 339.
12. Holland, H. J. and Medrud, R. C., *Journal of Applied Crystallography*, **10** (1977), 386.
13. Riedel, S., *Experimentelle Technik der Physik*, **31** (1983), 423.
14. Bakker, E. J., Stuart Frost, J. and Ogilvie, G. D., *Analytical Chemistry*, **42** (1970), 1117.
15. van den Belt, T. G. M. and Erkelens, H., *Computers and Chemistry*, **8** (1984), 169.
16. Meites, L., *Polarographic Techniques* (Interscience Publishers, New York, 1965), p. 411.
17. Stevens, C. L., Taylor, K. G., Munk, M. E., Marshall, W. S., Noll, K., Shah, G. D., Shah, L. G. and Uzu, K., *Journal of Medicinal Chemistry*, **8** (1965), 1.
18. Taylor, W. G. and Remers, W. A., *Journal of Medicinal Chemistry*, **18** (1975), 307.
19. Ploegmakers, H. H. J. L., Mertens, M. J. M. and van Oort, W. J., *Analytica Chimica Acta*, **174** (1985), 71.
20. Meites, L., *Polarographic Techniques* (Interscience Publishers, New York, 1965), p. 151.
21. Heyrovsky, J. and Ilkovic, D., *Collection of Czechoslovak Chemical Communications*, **7** (1935), 198.
22. Kolthoff, I. M. and Ligane, J. J., *Polarography* (2nd edn, Vol. I, Interscience Publishers, New York, 1952, p. 193.
23. Meites, L. *Treatise on Analytical Chemistry*, Ed. Kolthoff, I. M. and Elving, P.J. (part 1, Vol. 4, Interscience Publishers, New York, 1963), p. 2303.
24. den Hartigh, J., Driebergen, R. J., Nap, G., Engel, M. A. J. M., van Oort, W. J. and Hulshoff, A., *Bioelectrochemistry and Bioenergetics* (submitted).
25. Driebergen, R. J., Holthuis, J. J., den Hartigh, J., Blauw, J. S., van Oort, W. J., Hulshoff, A., Postma-Kelder, S. J., Verboom, W. and Reinhoudt, D. N., *Bioelectrochemistry and Bioenergetics* (submitted).

